# Small Extracellular Vesicles in Milk Cross the Blood-Brain Barrier in Murine Cerebral Cortex Endothelial Cells and Promote Dendritic Complexity in the Hippocampus and Brain Function in C57BL/6J Mice

**DOI:** 10.3389/fnut.2022.838543

**Published:** 2022-05-06

**Authors:** Fang Zhou, Pearl Ebea, Ezra Mutai, Haichuan Wang, Sonal Sukreet, Shya Navazesh, Haluk Dogan, Wenhao Li, Juan Cui, Peng Ji, Denise M. O. Ramirez, Janos Zempleni

**Affiliations:** ^1^Department of Nutrition and Health Sciences, University of Nebraska-Lincoln, Lincoln, NE, United States; ^2^Department of Nutrition, University of California, Davis, Davis, CA, United States; ^3^School of Computing, University of Nebraska-Lincoln, Lincoln, NE, United States; ^4^Department of Neurology, University of Texas Southwestern Medical Center, Dallas, TX, United States; ^5^Peter O’Donnell Jr. Brain Institute, University of Texas Southwestern Medical Center, Dallas, TX, United States

**Keywords:** blood brain barrier, gene expression, milk exosomes, neuronal development, serial two-photon tomography

## Abstract

Human milk contains large amounts of small extracellular vesicles (sEVs) and their microRNA cargos, whereas infant formulas contain only trace amounts of sEVs and microRNAs. We assessed the transport of sEVs across the blood-brain barrier (BBB) and sEV accumulation in distinct regions of the brain in brain endothelial cells and suckling mice. We further assessed sEV-dependent gene expression profiles and effects on the dendritic complexity of hippocampal granule cells and phenotypes of EV depletion in neonate, juvenile and adult mice. The transfer of sEVs across the BBB was assessed by using fluorophore-labeled bovine sEVs in brain endothelial bEnd.3 monolayers and dual chamber systems, and in wild-type newborn pups fostered to sEV and cargo tracking (ECT) dams that express sEVs labeled with a CD63-eGFP fusion protein for subsequent analysis by serial two-photon tomography and staining with anti-eGFP antibodies. Effects of EVs on gene expression and dendritic architecture of granule cells was analyzed in hippocampi from juvenile mice fed sEV and RNA-depleted (ERD) and sEV and RNA-sufficient (ERS) diets by using RNA-sequencing analysis and Golgi-Cox staining followed by integrated neuronal tracing and morphological analysis of neuronal dendrites, respectively. Spatial learning and severity of kainic acid-induced seizures were assessed in mice fed ERD and ERS diets. bEnd.3 cells internalized sEVs by using a saturable transport mechanism and secreted miR-34a across the basal membrane. sEVs penetrated the entire brain in fostering experiments; major regions of accumulation included the hippocampus, cortex and cerebellum. Two hundred ninety-five genes were differentially expressed in hippocampi from mice fed ERD and ERS diets; high-confidence gene networks included pathways implicated in axon guidance and calcium signaling. Juvenile pups fed the ERD diet had reduced dendritic complexity of dentate granule cells in the hippocampus, scored nine-fold lower in the Barnes maze test of spatial learning and memory, and the severity of seizures was 5-fold higher following kainic acid administration in adult mice fed the ERD diet compared to mice fed the ERS diet. We conclude that sEVs cross the BBB and contribute toward optimal neuronal development, spatial learning and memory, and resistance to kainic acid-induced seizures in mice.

## Introduction

Most cells synthesize and secrete nanoparticles called exosomes (∼100 nm) into the extracellular space (1). Exosomes travel to adjacent and distant recipient cells and play an important role in cell-to-cell communication (1), including transfer across the blood-brain barrier (BBB) (2, 3). Communication is achieved through the transfer of regulatory exosome cargos from donor cells to recipient cells as well as binding of exosomes to receptors on the recipient cell surface (1, 4). Among exosome cargos, microRNAs have gained particular attention because they regulate more than 60% of human genes and loss of microRNA biogenesis in Dicer knockout mice is embryonic lethal (5, 6). MicroRNAs are short non-coding RNAs that bind to complementary sequences in the 3′-untranslated regions in mRNAs (7, 8). If complementarity in the seed region (nucleotides 2-8 in microRNA) is perfect, mRNA is degraded (7, 8); if complementarity is imperfect, mRNA translation is halted (9, 10). In this paper we refer to the nanoparticles studied as small extracellular vesicles (sEVs) based on the rationale that their size matched that of exosomes but the ensosomal origin was not formally assessed (11).

We have pioneered a new line of discovery by demonstrating that sEVs and their microRNA cargos do not originate exclusively in endogenous synthesis but may also be absorbed from milk in human adults and neonate and adult mice and piglets (12–14). Evidence is accumulating that endogenous synthesis of microRNAs cannot compensate for dietary depletion of sEVs and their microRNAs cargos. We have developed sEV and RNA-depleted (ERD) and sEV and RNA-sufficient (ERS) diets based on the AIN-93G formulation and reported the content of sEVs and their cargos (15). Briefly, the pelleted diets contain a nutritionally relevant amount of bovine milk and the content of microRNAs and bioavailable sEVs is decreased by up to 99% and approximately 85% in the ERD diet compared to the ERS diet, respectively. All other nutrient are the same and the diets did not affect food and water intake and frequency, physical activity and variables of kidney and liver function in a previous study (16). The concentrations of microRNAs were up to 60% lower in the plasma, liver, skeletal muscle, intestinal mucosa and placenta in mice fed the ERD diet compared to controls fed the ERS diet (12, 16–19). The depletion of tissue microRNAs in mice fed ERD was associated with phenotypes such as altered purine metabolism, changes in bacterial communities in the gut, a moderate loss of muscle grip strength, increased severity of symptoms of inflammatory bowel disease and loss of fecundity and postnatal survival compared to ERS controls (16–21). sEV supplementation studies reported an increase in villus height and crypt depth in the murine intestinal mucosa, reduced severity of inflammation in mouse models of necrotizing enterocolitis and improved bone health in mouse models of osteoporosis compared to non-supplemented controls (22–24).

These observations are of great importance in nutrition, particularly the nutrition of infants. The American Academy of Pediatrics recommends that human milk be the sole source of nutrition in the first 6 months of life (25). Human milk contains large amounts of sEVs (2.2 × 10^11^/mL) loaded with more than 200 distinct microRNAs, whereas infant formulas are essentially free of sEVs and microRNAs (26). There may be implications of low sEV intake for the optimal neurological development of infants. For example, white matter, sub-cortical gray matter volume and cortical thickness were greater in breastfed infants compared with formula-fed infants although cause-and-effect relationships between sEV intake and brain development were not assessed (27). Only 26% of parents in the U.S. fed their infants exclusively with human milk in the first 6 months of life in 2018 (28). The 2.8 million infants born annually in the U.S. that are partially or exclusively formula-fed do not realize the potential benefits conferred by sEVs (29).

Previously, we provided evidence that a large percentage of orally administered sEVs and microRNAs cargos in milk accumulate in the brain in suckling mice and piglets and adult mice (14). These studies did not formally exclude the possibility that the sEVs remained in the vasculature. In this paper we investigated the transport of milk sEVs across the BBB and sEV accumulation in distinct regions of the brain, sEV-dependent gene expression profiles and functional effects such as neuronal development and brain phenotypes of sEV depletion in cell culture models and mice. Studies of neuronal development focused on dendritic complexity because dendritic arborization and branching patterns are susceptible to modulation of environmental cues (30). Exosomes are implicated in intercellular communication among neurons. For example, the injection of exosomes from neural cell cultures into the lateral ventricles of postnatal day 4 mice increased neural proliferation enhanced in dentate gyrus (31). Motivated by this prior knowledge, we assessed the contribution of sEVs in milk toward optimal neuronal development and brain health in mice.

## Materials and Methods

### Isolation and Labeling of Small Extracellular Vesicles

Bovine milk sEVs (BEVs) were isolated from skim milk from a local grocery store by using sequential ultracentrifugation and authenticated by using Nanosight NS300 nanoparticle size analysis, scanning electron microscopy and transmission electron microscopy as previously described (Supplementary Figure 1) (32). The antibodies and their dilutions used in immunoblot analysis were the same as previously described (32). Protocol details were deposited in the EV-Track database (ID EV210338). BEVs were suspended in sterile phosphate-buffered saline (PBS) and kept at −80°C until use. For transport studies in cell monolayers, BEVs were labeled with FM 4-64 (Molecular Probes, Inc., Eugene, OR, United States) or by labeling RNA cargos by using the ExoGlow-RNA™ EV Labeling Kit (System Biosciences, Inc., Palo Alto, CA, United States) following the manufacturers’ recommendations. For transport studies in dual-chambers, BEVs were loaded with synthetic IRDye-labeled miR-34a as previously described (14).

### Bovine Milk Small Extracellular Vesicle Transport in Cell Cultures

Murine brain endothelial bEnd.3 cells [American Type Culture Collection (ATCC) CRL-2299, passages 21 – 30)] and C8-D1A astrocytes (ATCC CRL-2541, passage unknown to ATCC) were purchased form ATCC. BV2 microglia (passage 15 – 25) were a gift from Dr. Sanjay Maggirwar (University of Rochester Medical Center, Rochester, NY, United States). Cells were cultured following ATCC recommendations. In monolayer studies, uptake of BEVs by bEnd.3 cells and BV2 microglia was assessed as previously described using times, concentrations and competitors shown in Results (13). Transport kinetics was modeled using the Michaelis–Menten equation and non-linear regression; modeling was conducted using GraphPad Prism 6.0 (GraphPad Software). Confocal Z-stacks were collected at 60-fold magnification using 300 nm z-spacing on an A1R-Ti2 confocal system (Nikon) and used to determine whether bEnd.3 cells internalized BEVs or whether BEVs adsorbed to the cell surface. Dual chamber assays as a model of transport across the blood-brain barrier (BBB) were conducted as previously described with the following modifications (13). bEnd.3 cells were seeded on the semiporous membrane in co-culture with astrocytes in the bottom chamber; the integrity of the bEnd.3 cell monolayer was assessed by using trans endothelial electrical resistance in an Epithelial Volt/Ohm meter equipped with STX2 electrodes (EMD Millipore Corporation). Uptake of BEVs labeled with FM 4-64 and RNA cargos labeled with ExoGlow-RNA™ was quantified by using a microplate fluorescence reader (BioTek Instruments, Inc., Winooski, VT, United States) and confocal microscopy imaging, respectively. The transfer of IRDye-labeled miR-34a across a bEnd.3 cell monolayer was measured in dual chamber assays by using an Odyssey^®^ imaging system (LI-COR, Inc., Lincoln, NE, United States).

### Small Extracellular Vesicle Distribution in Regions of the Mouse Brain

We developed an sEV and cargo tracking (ECT) mouse on the C57BL/6J genetic background that enables studies of exosome and cargo trafficking among tissues, as well as studies of the transfer of sEVs from dam to pup (14). Briefly, ECT mice express an open reading frame (ORF) coding for the exosome marker, CD63 (33) fused with enhanced green fluorescent protein (eGFP) flanked by loxP sites. In the presence of cre recombinase, the CD63-eGFP ORF is removed, and mice express an open reading frame coding for a fusion protein of CD63, near-infrared protein (iRFP), transmembrane domain and a second iRFP. The second iRFP localizes to the outer exosome surface and can be used to collect exosomes for cargo analysis.

Wild-type (WT) newborn C57BL/6J pups were fostered to ECT dams or WT dams from synchronized pregnancies and nursed for 17 days. Pups were euthanized and brains were fixed *via* transcardial perfusion of 4% paraformaldehyde, stored in phosphate-buffered saline and shipped to the Whole Brain Microscopy Facility at the University of Texas Southwestern Medical Center for analysis by serial two-photon tomography (STPT) (34–37), and immunostaining of brain slices with anti-GFP antibodies and confocal imaging. Eleven whole brains from WT pups fostered to ECT dams across three separate litters and two brains from WT pups fostered to WT dams were used for STPT and anti-GFP immunostaining of isolated coronal brain sections. STPT is a high-resolution, high-throughput volumetric imaging strategy for assessing the regional distribution of native fluorescent labels throughout entire uncleared mouse brains *via* serial vibratome sectioning and mosaic two-photon imaging (34–37). Vibratome sections in the coronal plane at 75 μm thickness were generated *via* STPT. A subset of the sections were selected for immunoamplification of the native GFP signals *via* immunostaining with anti-GFP antibodies (Invitrogen # A11122; 1:500 dilution) and confocal imaging. Two sections (one at the level of the dorsal hippocampus and one at the level of the cerebellum) were selected from each brain for immunostaining and confocal imaging. Two 20X fields of view, at the cortex and hippocampal dentate gyrus, were acquired from the rostral section and one 20X field of view including cerebellar molecular, Purkinje and granule cell layers was acquired from the caudal section. Immunostained coronal brain sections were imaged using a Zeiss LSM 780 confocal microscope (Live Cell Imaging Facility, UTSW) at 20X and with the same acquisition parameters across samples. All animal studies in this paper were approved by the Institutional Animal Care Program at the University of Nebraska-Lincoln (protocols 1229 and 1713).

### Gene Expression Analysis

We assessed BEV-dependent gene networks in the left hippocampus in male and female C57BL/6J mice (Jackson Labs., stock 000664). Briefly, C57BL/6 mice were fed ERD or ERS diets starting at age 3 weeks for 7 weeks when mice were mated (15). Pups born to these breeders were continued on parental diets until age 7 weeks. Pups were euthanized *via* transcardial perfusion with phosphate-buffered saline and brains were excised. The left hippocampus was dissected and flash frozen in liquid nitrogen for storage at −80°C. Total RNA was extracted by using miRNeasy Kit (Qiagen, Inc., Hilden, Germany) according to the manufacturer’s instructions, and the concentration, quality and integrity of RNA was analyzed as previously described (38, 39). cDNA libraries were prepared by using a proprietary kit and sequenced by using a paired-end 150 base-pair protocol and the NovaSeq platform (Illumina, Inc., San Diego, CA, United States) in the Beijing Genomic Institute. RNA-seq data were analyzed as previously described (16). RNA-seq data were validated for seven differentially expressed genes by using quantitative real-time PCR (qRT-PCR) with primers shown in Supplementary Table 1 (40–47). Briefly, total RNA was reverse transcribed using the QuantiTec Reverse Transcription kit (Qiagen, Inc., Hilden, Germany) and qPCR was performed with SsoAdvanced Universal SYBR Green Supermix in a 10-μl reaction volume by following the manufacturer’s instructions (Bio-Rad Laboratories, Inc., Hercules, CA, United States) using the following conditions: 94°C for 3 min, followed by 40 cycles at 94°C for 30 s, 60°C for 30 s, and 72°C for 30 s. The relative gene expression were calculated using 2^–ΔΔ^Ct method with GAPDH as internal reference gene. Three biological repeats were analyzed and each biological repeat was analyzed three times.

### Dendritic Complexity of Dentate Granule Cells

Neuronal dendrites are highly branched, tree-like structures, the morphological complexity of which is linked to signal integration and firing pattern of individual neurons and the functionality of neural circuitry (48). Mice were fed ERD and ERS diets as described above. Hippocampal neurons from the left hemispheres of mice were stained using the Golgi-Cox method as previously described (49, 50). Brightfield images of dentate granule cells in the suprapyramidal blade were collected in the Z plane at 20× magnification in 0.3-μm steps with an Olympus IX-81 inverted spinning disk confocal microscope using MetaMorph Advanced software version 7.1 (Molecular Devices). Three-dimensional dendritic structures of 3 – 5 granule cells were manually traced and reconstructed in each hippocampus using NeuroLucida version 2019.1.2 (MBF Bioscience). Dendritic complexity and other morphological features were quantified *via* Sholl analysis through NeuroLucida Explorer version 2019.1.2 (MBF Bioscience). Research staff was blinded regarding the treatment of mice.

### Phenotyping Studies

Phenotyping studies focused on the assessment of spatial learning and memory (SLM), kainic acid-induced seizures, acoustic startle response and prepulse inhibition. These endpoints were chosen because the hippocampus has been implicated in SLM, kainic acid-induced seizures and acoustic startle response in mice and rats (51–53). The choice of phenotypes is also consistent with our observations that milk sEVs accumulated in the hippocampus (in addition to other regions) and altered the expression of genes implicated in axon guidance and calcium signaling in murine hippocampi in mice; dietary depletion of sEVs led to a decrease in neuronal branching in murine dentate granule cells (see the section “Results”).

Spatial learning and memory was assessed at two ages by using the Barnes maze (54). The Barnes maze measures the ability of a mouse to learn and remember the location of an escape hole on a circular surface with the help of a visual cue; low values represent strong test performance (54). Mice were trained on six consecutive days prior to measuring the time needed to locate the escape hole on day 7. Training and measurement were stopped after 5 min if a mouse failed to enter the escape hole. Measurements were take by using the ANY-maze video tracking system (Stoelting Co., Wood Dale, IL, United States). SLM experiments were conducted using the same mice that were subsequently used in RNA-sequencing analysis, except that additional mice were included in tests of SLM. SLM was also assessed in adult mice ages 12 – 15 weeks fed ERD and ERS diets starting at age 3 weeks. Mice were randomly assigned to diet groups and both sexes were studied.

In tests of seizure severity, C57BL/6J mice ages 3 weeks were fed ERD or ERS diets for 18 weeks when seizures were triggered by subcutaneous administration of kainic acid (25 mg/kg body weight) (55). Kainic acid is a non-hydrolyzable glutamate analog that binds to five glutamate receptors in the brain (56, 57), thereby causing neuronal excitotoxicity and seizures (55). The severity of seizures was scored using a modified Racine scale (58, 59). In the modified Racine scale, seizures are scored at timed intervals for 2 h and the highest score in each 5-min block are reported: 0, no seizure; 1, immobility; 2, forelimb and/or tail extension; 3, automatisms; 4, forelimb clonus, rearing, and/or falling; 5, repetition of stage 4; 6, tonic–clonic seizures; and 7, death.

Acoustic startle response and pre-pulse inhibition were assessed as previously described using the mice from the Barnes maze experiments 1 day after conducting the studies in the maze (60, 61). Pre-pulse inhibition of the acoustic startle response was measured using prepulses of 68, 74, and 80 dB for 10 ms with 65-dB background white noise, followed by a pulse of 105 dB for 20 ms; intervals between stimuli were random 10 – 30 ms. The percent prepulse inhibition of acoustic startle response was calculated as [(1 – (startle response at 105 dB with prepulse stimuli)/startle response for startle response at 105 dB without prepulse)] × 100. The acoustic startle response was scored ten times per mouse for each of the following conditions: 105-dB startle stimulus without prepulse, 105-dB startle stimulus with prepulses of 68, 74, 80 dB, and no startle stimulus with pulses of 80 dB. Test were performed by using an SR-LAB Startle Response System (San Diego Instruments, San Diego, CA, United States).

### Statistical Analysis

The *F*-test was used to assess the homogeneity of variances (62). Some variances were heterogeneous, e.g., Racine scale scores. Log transformation of these data resulted in homogenous data variation. Data from time-dependent transwell studies were analyzed by using repeated measures one-way ANOVA followed by Dunnett’s multiple comparisons test. The Kruskal-Wallis test was used for the analysis of data from acoustic startle response experiments. Data from both seizure studies and the Sholl analysis were analyzed by using repeated measures ANOVA and mixed procedure. The distance to soma was used as the within-subjects repeated measure. qRT-PCR data were analyzed by *t*-test. The model includes treatment as the fixed effect and mouse nested in treatment as the random term. Data analysis was conducted by using SPSS 27, SAS 9.4 and GraphPad Prism 9.0. *P* < 0.05 was considered statistically significant. Data are reported as mean ± SEM.

## Results

### Transport of Small Extracellular Vesicles by Brain Cells

**FIGURE 1:**
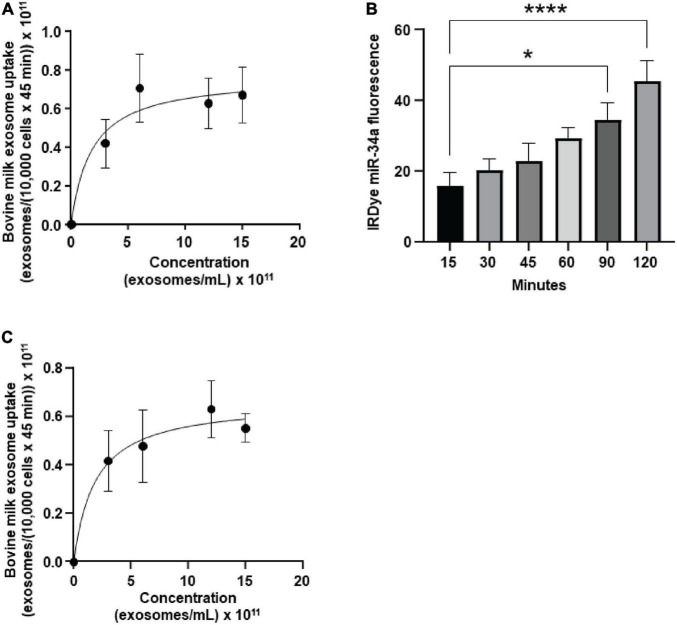
Transport of bovine milk small extracellular vesicles (BEVs) by murine brain cells. **(A)** Saturation kinetics of BEV uptake by bEnd.3 cells. **(B)** Secretion of IRDye-labeled miR-34a, loaded into BEVs, in a dual chamber assay. **(C)** Saturation kinetics of BEV uptake by murine BV2 microglia. Values are means ± SEMs, *n* = 3. *P < 0.05; ****P < 0.001.

bEnd.3 cells internalized BEVs by using a saturable process and secreted microRNA cargos across the basal membrane. The uptake of FM4-64-labeled BEVs was modeled using the Michaelis-Menten equation (Figure 1A): transporter capacity (maximal velocity, V_max_) = 0.77 ± 0.18 × 10^11^ BEVs/(10,000 cells × 45 min) and affinity (Michaelis–Menten constant, K_m_) = 1.8 ± 2.0 × 10^11^ BEVs/mL). All subsequent studies in bEnd.3 cells were conducted under conditions when BEV concentrations (6 × 10^11^ BEVs/mL) and incubation times (45 min) do not limit BEV uptake (Supplementary Figure 2). Z-stack confocal imaging confirmed that bEnd.3 cells internalized ExoGlow-RNA™ labeled BEVs, as opposed to BEVs adsorbing to the cell surface (Supplementary Figure 3). Upon internalization, the ExoGlow-RNA™ labeled BEVs localized to the cell cytoplasm (Supplementary Figure 4).

In a dual chamber model of transfer across the BBB, bEnd.3 cells secreted IRDye-labeled miR-34a, loaded into BEVs, across the basal membrane into the bottom chamber in co-cultures with astrocytes (Figure 1B). The integrity of the bEnd.3 cell monolayer on the semiporous membrane was assessed by measuring the *trans* endothelial electrical resistance and reached a plateau approximately 4 days after seeding the cells (Supplementary Figure 5); absence of astrocytes in the bottom chamber caused a loss of monolayer integrity. BEVs that crossed the BBB, were internalized by brain cells, using brain macrophages, BV2 microglia as model. BEVs uptake by BV2 microglia followed saturation kinetics (Figure 1C): V_max_ = 0.66 ± 0.14 × 10^11^ BEVs/(10,000 cells × 45 min) and K_m_ = 1.9 ± 1.9 × 10^11^ BEVs/mL.

### Small Extracellular Vesicle Distribution in the Brain

**FIGURE 2:**
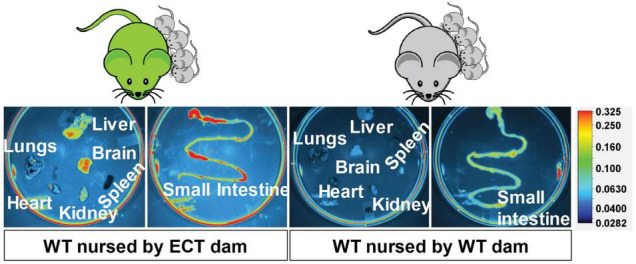
Accumulation of eGFP-positive milk sEVs in peripheral tissues and the small intestinal mucoca in wild-type pups fostered to ECT dams and nursed for 17 days. Wild-type pups fostered to wild-type dams served as controls.

**FIGURE 3:**
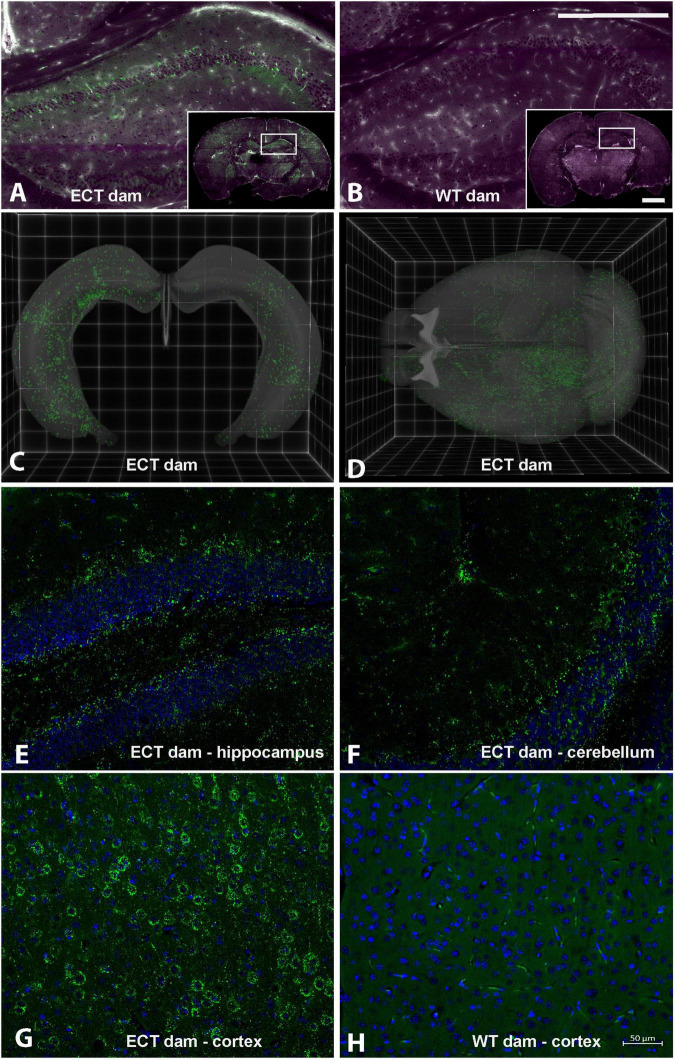
Serial Two-Photon Tomography (STPT) and confocal imaging in brains from wild-type pups fostered to either ECT or wild-type dams. **(A)** Single 2D section at the level of the dorsal hippocampus from a wild-type mouse pup fostered to an ECT dam acquired using STPT. Inset, whole coronal section with right hippocampus indicated with white box. Accumulation of GFP positive sEVs (green) is apparent in hippocampus and other areas throughout the section. Tissue autofluorescent signal is shown in magenta. **(B)** Single 2D section at the level of the dorsal hippocampus from a wild-type mouse pup fostered to a wild-type dam acquired using STPT. Inset, whole coronal section with right hippocampus indicated with white box. Accumulation of GFP positive sEVs was not detected. Tissue autofluorescent signal is shown in magenta. **(C)** 3D rendering of bilateral hippocampal volumes from a wild-type mouse pup fostered to an ECT dam acquired using STPT. Native GFP signal indicative of sEV accumulation is present throughout the entire hippocampal volume. **(D)** 3D rendering of entire brain volume from a wild-type mouse pup fostered to an ECT dam acquired using STPT. Native GFP signal indicative of sEV accumulation is present in many regions throughout the brain. **(E–H)** Confocal images from isolated coronal sections immunostained with anti-GFP antibodies, shown in green, and DAPI as nuclear counterstain, shown in blue. Images in panels **(E–G)** are from sections from the brain of a wild-type mouse pup fostered to an ECT dam and show accumulation of GFP positive sEVs in the hippocampus, cerebellum and cortex, respectively. Panel **(H)** shows an image from the cortex of the wild-type mouse pup fostered to a wild-type dam and no GFP signal was observed. Scale bars in panel **(B)** apply to panels **(A,B)**: main panel = 750 μm, inset = 2.25 mm. Scale bar in panel **(H)** applies to panels **(E–H)**.

eGFP-positive sEVs accumulated primarily in the brain but also in the liver and small intestinal mucosa in WT pups fostered to ECT dams (Figure 2). No eGFP fluorescence was detected in the brain and liver in WT pups fosterd to WT dams, and the signal in the small intestine represents autofluorescence (control). Brains from WT pups fostered to either ECT or WT dams were subjected to serial two-photon tomography and anti-GFP immunostaining in isolated coronal brain sections. Accumulation of GFP labeled sEVs was evaluated in cortex, hippocampus and cerebellum using confocal imaging. Out of 11 brains from pups fostered to ECT dams across three litters, we observed positive anti-GFP staining in at least one of these three regions in seven of the brains. In total, brains from seven male pups and 4 female pups fostered to ECT dams were imaged. 2/7 male and 2/4 female brains did not show positive GFP signal. GFP signal was not observed in two brains from WT pups fostered to WT dams (one male and one female). Representative 2D coronal section images at the level of the dorsal hippocampus from the brain of a WT pup fostered to an ECT dam (Figure 3A) and that of a WT pup fostered to a WT dam (Figure 3B) shows robust accumulation of GFP labeled sEVs throughout the section from the brain of the pup which was fostered to the ECT dam (Figure 3A), but no GFP signal was observed in the pup fostered to the WT dam (Figure 3B). 3D renderings of the hippocampus (Figure 3C) and the entire brain (Figure 3D) from the pup fostered to the ECT dam demonstrate robust accumulation of GFP labeled exosomes throughout the hippocampus and many other brain regions. Figures 3E–G show confocal imaging of anti-GFP immunostaining in the brain of a different WT pup fostered to an ECT dam in the hippocampus, cerebellum and cortex. Positive GFP labeling was observed in each of these three brain regions. GFP labeled sEVs were detected in the hippocampal granule cell layer in the dentate gyrus (Figure 3E). In contrast, no GFP labeling was seen in brains of pups fostered to WT dams as shown in Figure 3H.

### Bovine Milk Small Extracellular Vesicle-Dependent Biological Pathways

**FIGURE 4:**
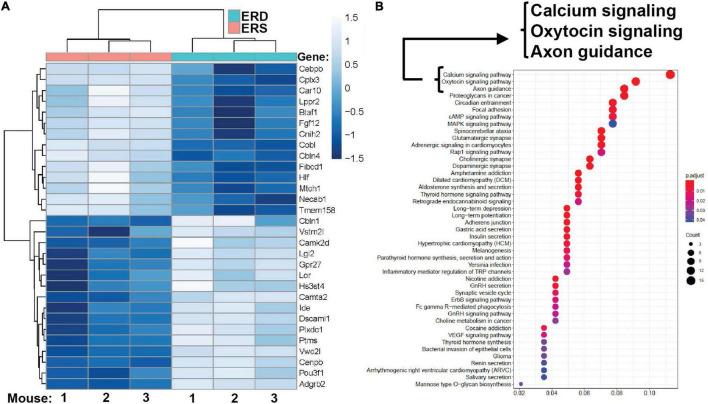
Gene expression. **(A)** Top 30 differentially expressed genes in the left hippocampus in male pups. Means without a common letter differ (*P* < 0.05; *q* < 0.3; *n* = 3). **(B)** KEGG pathways. KEGG, Kyoto Encyclopedia of Genes and Genomes.

Dietary intake of BEVs altered gene expression in the brain. Two hundred ninety-five genes were differentially expressed in the left hippocampus in male mice fed ERD or ERS diets for 7 weeks (Figure 4A). Three genes (*Dcn*, decorin; *Nos1*, nitric oxide synthase 1; *Ndn*, necdin) were differentially expressed in females. Raw sequencing data can be accessed in the NCBI-BioProject database through accession number PRJNA783128. KEGG pathway analysis revealed 45 BEV-dependent biological pathways in males, each with at least three BEV-dependent genes (Figure 4B). Pathways of calcium signaling and axon guidance were among the three top-ranked pathways. No KEGG pathways emerged in the analysis of mRNA expression data in females.

qRT-PCR analysis of mRNA expression in hippocampi confirmed the patterns obtained by RNA-sequencing analysis (Supplementary Figure 6). For example, the expression of *BTAF1* and *Dscaml1* was significantly higher and lower, respectively, in males fed the ERS diet compared to males fed the ERD diet in both qRT-PCR analysis and RNA-sequencing analysis. Expression of *Ndn*, *Nos2* and *Dcn* was lower in females fed the ERS diet compared to females fed the ERD diet in both qRT-PCR analysis and RNA-sequencing analysis. Expression of *Cplx3* and *Gpr27* was significantly higher and lower, respectively, in males fed the ERS diet compared to males fed the ERD diet in both qRT-PCR analysis and RNA-sequencing analysis but diet effects were not statistically significant in qRT-PCR analysis.

### Phenotypes of Bovine Milk Small Extracellular Vesicle Depletion

**FIGURE 5:**
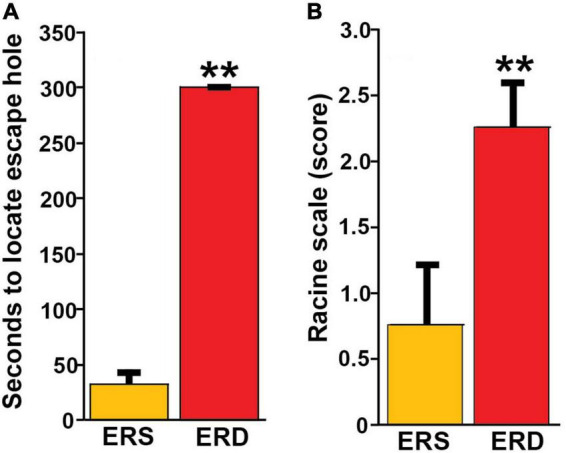
Effects of BEV-defined diets on brain function in mice. **(A)** SLM in female pups, age 4 weeks. Values are means ± SEMs (**P < 0.01, *n* = 5). Means without a common letter differ. **(B)** Seizure activity 30 min after kainic acid administration in male mice ages 21 weeks. Values are means ± SEMs (**P < 0.01, *n* = 8).

Bovine milk small extracellular vesicle-dependent changes in gene expression and neuronal growth were associated with impaired SLM and increased the severity of kainic acid-induced seizures in mice fed the ERD diet compared to mice fed the ERS diet. For example, when female pups were nursed by dams fed the ERS diet for 3 weeks and continued on the maternal diet for 1 week, the mice performed nine times better in the Barnes maze test of SLM compared to mice nursed by ERD dams and fed the ERD diet for 1 week (Figure 5A). Diet effects on SLM were more modest in males than in females and in mice older than 4 weeks (Supplementary Figure 7); the only exception are females ages 7 weeks in which diet effects were similar to females ages 4 weeks. Path length patterns matched the time it took mice to locate and enter the escape hole (Supplementary Figure 7). Beneficial effects of BEVs on brain function were not limited to SLM but extended to seizure activity which was five times higher in male mice fed the ERD diet 30 min after administration of kainic acid compared to ERS controls (Figure 5B and Supplementary Table 2). Note that the effect of diets on kainic acid-induced seizure activity were also detectable in females but effects were modest when compared to males and not statistically significant (Supplementary Table 2). Dietary effects on pre-pulse inhibition of acoustic startle response were modest and not statistically significant in most age groups and both sexes (data not shown).

### Dendritic Complexity

**FIGURE 6:**
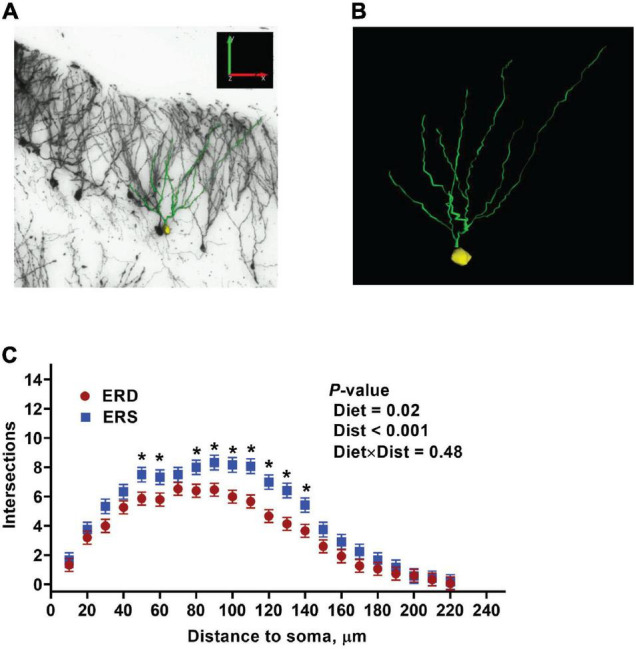
Three-dimensional dendritic architecture **(A,B)** and Sholl analysis of dendritic complexity **(C)** of dentate granule cells from murine hippocampus (*n* = 4 – 5 mice; 3 – 5 granule cells per mouse).

Given the preliminary evidence of neuronal accumulation of BEV-derived microRNAs, as well as alterations in hippocampal pathways implicated in neuronal development and cognitive deficits in SLM after dietary BEV depletion, we evaluated its impact on dendritic complexity of dentate granule cells in the hippocampus. Representative samples of traced three-dimensional dendritic architecture of dentate granule cells were shown in Figures 6A,B. Sholl analyses evaluated dendritic complexity by quantifying the number of interactions between dendrites and soma-oriented concentric spheres of increasing diameters (Figure 6C). There is a significant main effect of diet on dendritic complexity. This is evidenced by the smaller number of dendritic intersections in DG neurons from mice fed the ERD diet compared to mice fed the ERS diet (*P* < 0.05), suggesting that milk sEV deficiency resulted in underdevelopment of neuron dendritic architecture in the developing brain. This difference was primarily due to a greater number of branch nodes of granule cells in ERS mice compared to ERD mice (Table 1). In addition, there was a trend toward a greater number of dendritic tips in ERS mice compared to ERD mice (*P* = 0.08). The number of primary dendrites and total dendritic length of dentate granule cells were not significantly different between diet groups (Table 1).

**TABLE 1:** Effects of small extracellular vesicles (sEVs) on dendritic morphology of dentate granule cells in murine hippocampus (mean ± SEM).

	ERD	ERS	*P*-value
Primary dendrite, n	2.0 ± 0.2	1.9 ± 0.2	0.73
Branch node, n	6.9 ± 0.8	9.8 ± 0.8	0.04
Branch tip, n	9.0 ± 0.9	11.6 ± 0.9	0.08
Total dendritic length, μm	913.9 ± 76.5	1099.2 ± 77.1	0.13

## Discussion

This is the first report that orally ingested sEVs from milk are transported across the BBB and accumulate in distinct regions of the brain. This report also provides experimental evidence that sEVs deliver messages that alter gene expression and promote neuronal growth in the brain, and dietary depletion of sEVs and cargos in milk elicits phenotypes such as impaired SLM and increased severity of kainic acid-induced seizures. These discoveries are of great significance in infant nutrition because of the substantially greater content of sEVs and microRNA cargos in human milk compared to infant formulas, and milk is the sole source of nutrition in the first stages of mammalian life (25, 26). Neurological phenotypes of sEV depletion were evident in mice in this study, but causal relationships between the consumption of sEV-poor formulas and sEV-rich human milk in the neurological development of infants have yet to be investigated. There is circumstantial evidence in support of the theory that sEVs in milk might contribute toward optimal brain development in infants, although the studies were not designed to look specifically at sEVs and, therefore, other compounds in human milk could also have contributed to the positive effects of breastfeeding. For example, breastfed infants scored higher than formula-fed infants in tests of mental and psychomotor development, and breastfeeding increased white matter, sub-cortical gray matter volume and cortical thickness in infants (27, 63). Breastfed infants scored higher in cognition tests than formula-fed infants in a meta-analysis (64). To put the brain phenotypes reported in this study in context, when mice were fed n-3 polyunsaturated fatty acid (PUFA)-defined diets known to improve cognitive function, mice fed an n-3 PUFA-sufficient diet performed 1.35-fold better in the Barnes maze compared to mice fed an n-3 PUFA-deficient diet (65), whereas in our study, mice fed the ERS diet performed nine times better than the mice fed the ERD diet on the Barnes maze.

Confidence in the data reported here is high, because sEVs accumulated and altered gene expression and dendritic architecture in the hippocampus, which is implicated in SLM, kainic acid-induced seizures and pre-pulse inhibition of the acoustic startle response in mice (51, 53). We note that the presence of GFP positive sEVs was consistently observed in the hippocampal granule cell layer among animals with detectable levels of GFP-labeled ME accumulation (Figure 3 and data not shown), where reduced dendritic complexity was seen after dietary exosome depletion (ERD vs. ERS diet treatment; Figure 6). Also, there is a degree of specificity to the neurological phenotypes associated with sEV depletion. For example, effects of BEV depletion on muscle grip strength were modest in previous studies in mice and rats and this study revealed modest effects in rotarod and startle response tests (16, 21). The exact mechanism of action by which BEV depletion impairs SLM and increases the severity of kainic acid-induced seizures remains elusive. If neurological phenotypes are caused by a depletion of microRNA cargos, then miR-30d and let-7b might be the prime candidates for facilitating the phenotypes. MicroRNA 30d and let-7b are the two most abundant microRNAs in sEVs in human milk and loss of miR-30d and let-7b signaling impaired axonal outgrowth in early neuronal development (26, 66).

Importantly, our results indicated that GFP-labeled sEV accumulation was not apparent in a subset of the wild-type mouse pups fostered to ECT dams. Differences in milk intake, absorption and integrity of the BBB might explain why milk sEVs accumulated at a detectable level in some, but not all, mice in this study. We reported a similar phenomenon in wild-type piglets fostered to sows that express extracellular vesicles endogenously labeled with ZsGreen1 in milk (14). Future studies will continue to investigate the underlying bases for observed inter-individual differences in sEV accumulation across brain regions.

This study suggests that some neurological effects of BEV depend on sex and age. For example, the number of BEV-dependent genes in the hippocampus was 40 times greater in males than in females ages 7 weeks, and the effect of BEV depletion on SLM was 1.7 times stronger in males than females ages 15–18 weeks. As for age effects, examples include that BEV depletion had a stronger effect on SLM in female than male mice ages 4 weeks whereas effects of BEV depletion on SLM were stronger in male than female mice ages 15–18 weeks. We do not have to offer an explanation for the sex effects observed. There is precedent for effects of age as it interfaces with brain development and nutrition, e.g., the rate of gray matter accumulation peaked 1 or 2 years earlier in female than male adolescents, and maternal diet during pregnancy affected gene expression in male fetal brain more than in females in a mouse model of obesity (67, 68).

Dietary depletion of milk sEVs elicited phenotypes of depletion in both males and females whereas sEV-dependent KEGG were identified only in males. This discordance may be explained as follows. First, a mutation in a single gene can have profound effects on brain function, e.g., a mutation in the *SynGAP1* gene causes *Syngap1* haploinsufficiency leading to neurodevelopmental disorders defined by autistic traits, cognitive impairment and epilepsy (69). Second, the dietary intake of milk sEVs causes changes in the gut microbiome which are associated with changes in the levels of neurotransmitters such as purines, L-glutamate and tryptophan metabolites in human and murine hosts (17, 20, 70).

Note that bovine milk was used to prepare ERD and ERS diets and the diets were fed to mice, i.e., one might wonder about species effects. As discussed above, the content of microRNAs and bioavailable sEVs is decreased by up to 99% and approximately 85% in the ERD diet compared to the ERS diet, respectively whereas all other nutrients are the same (15). The nucleotide sequences of microRNAs is highly conserved among mammals and we therefore propose that alterations in the intake of BEVs is responsible for the phenotypes observed in mice (71). A possible alternative explantation is gene regulation by non-coding RNAs other than microRNAs in milk sEVs, because they accounted for more than 98% of the sequencing reads in sEVs from human milk (72). The contribution of no-coding RNAs other than microRNAs to phenotypes observed in ERD and ERS feeding studies remains to be experimentally tested.

This report, in conjunction with previous studies of the bioavailability and phenotypes of milksEV and their microRNA cargos, suggests that milk sEVs and microRNA cargos meet the definition of bioactive compounds by the National Cancer Institute which is “A type of chemical found in small amounts in plants and certain foods (such as fruits, vegetables, nuts, oils, and whole grains) which has actions in the body that may promote good health” (73). Taken this theory one step further, conditionally essential nutrients “can usually be synthesized in adequate amounts endogenously, but may require exogenous supplementation during some circumstances” (74). Future lines of investigation will further delineate the roles of sEVs and their miRNA cargos in neurodevelopment. For example, it will be important to determine whether our findings in mice translate into human populations, particularly infants. One could consider assessing neurological function in cohorts of infants fed sEV-poor formulas and sEV-rich human milk. Such studies will inform stakeholders whether the addition of sEVs to infant formulas warrants consideration. Along these lines it will be important to assess whether phenotypes of sEV depletion in infancy persist post weaning. Future studies will also need to fill knowledge gaps as to what cargos in milk sEVs elicit neurological phenotypes and what signaling compounds these cargos affect.

## Data Availability Statement

The datasets presented in this study can be found in online repositories. The names of the repository/repositories and accession number(s) can be found below: https://www.ncbi.nlm.nih.gov/, PRJNA783128.

## Ethics Statement

The animal study was reviewed and approved by the Institutional Animal Care Program, University of Nebraska-Lincoln.

## Author Contributions

FZ performed the experiments, analyzed the data, performed the statistical analysis, and the manuscript revision. PE performed the experiments, analyzed the data, performed the statistical analysis, and drafting of the manuscript. EM and SN performed the experiments, analyzed the data, and performed the statistical analysis. SS and HW contributed to the experiments. HD contributed to the experimental design, analyzed the data, and interpreted the data. WL performed the experiments. JC, PJ, and DR contributed to the experimental design, analyzed the data, interpreted the data, and performed manuscript revision. JZ contributed to the experimental design, wrote the manuscript, and took responsibility for the final content. All authors read and approved the final manuscript.

## Conflict of Interest

JZ serves as consultant for PureTech Health, Inc., (Boston, MA). The remaining authors declare that the research was conducted in the absence of any commercial or financial relationships that could be construed as a potential conflict of interest.

## Publisher’s Note

All claims expressed in this article are solely those of the authors and do not necessarily represent those of their affiliated organizations, or those of the publisher, the editors and the reviewers. Any product that may be evaluated in this article, or claim that may be made by its manufacturer, is not guaranteed or endorsed by the publisher.

## Abbreviations

BBB: blood-brain barrier
BEV: bovine milk small extracellular vesicle
ECT: small extracellular vesicle and cargo tracking
eGFP: enhanced green fluorescent protein
ERD: small extracellular vesicle and RNA-depleted
ERS: small extracellular vesicle and RNA-sufficient
iRFP: near-infrared protein
KEGG: Kyoto Encyclopedia of Genes and Genomes
ORF: open reading frame
PBS: phosphate-buffered saline
PUFA: n-3 polyunsaturated fatty acid
sEV: small extracellular vesicle
qRT-PCR: quantitative real-time polymerase cgain reaction
TEER: transepithelial electrical resistance.
