# Supplementary material for: Small Extracellular Vesicles in Milk Cross the Blood-Brain Barrier in Murine Cerebral Cortex Endothelial Cells and Promote Dendritic Complexity in the Hippocampus and Brain Function in C57BL/6J Mice
Source: Front Nutr. 2022 May 6;9:838543. doi: 10.3389/fnut.2022.838543 (PMC9121399; doi:10.3389/fnut.2022.838543)
Supplement: Supplementary file 1 [file Data_Sheet_1.PDF]

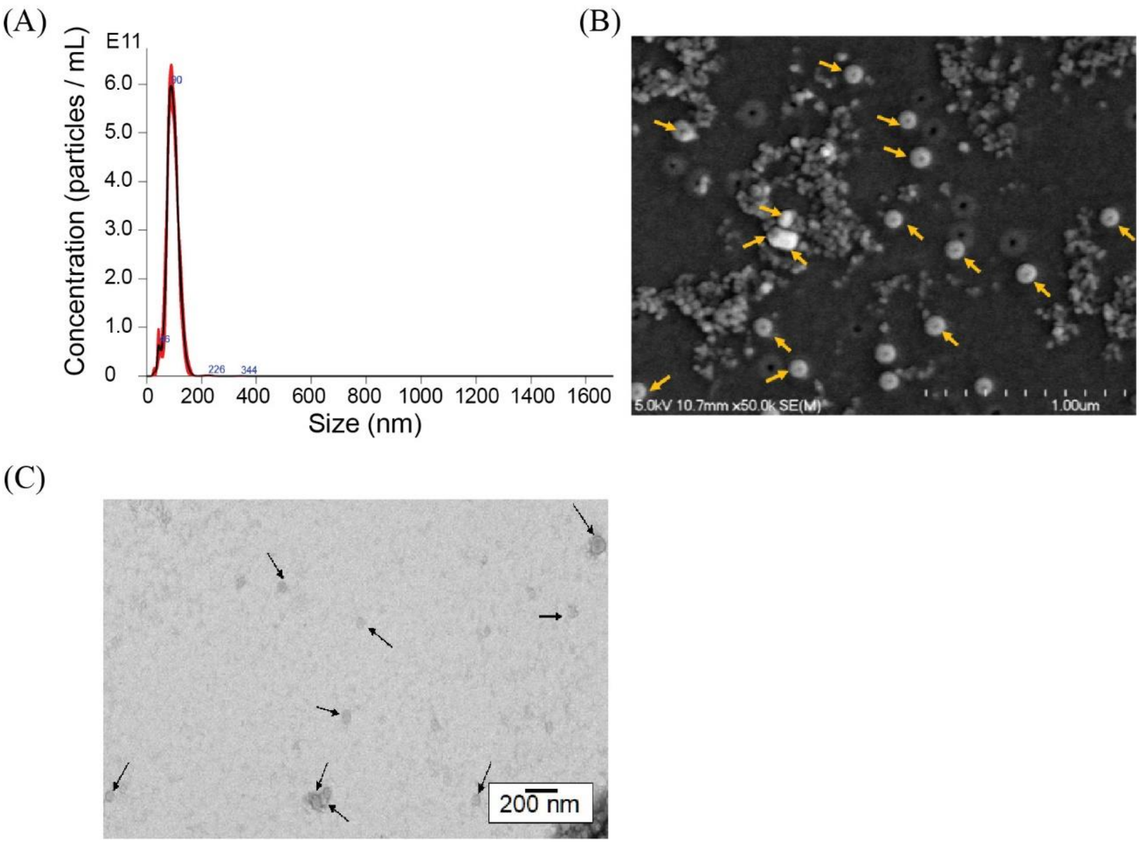

Supplementary Fig. S1. BEVs were authenticated by nanoparticle size analysis **(A)**, scanning electron microscopy **(B)** and transmission electron microscopy **(C)**. Arrows point toward BEVs.

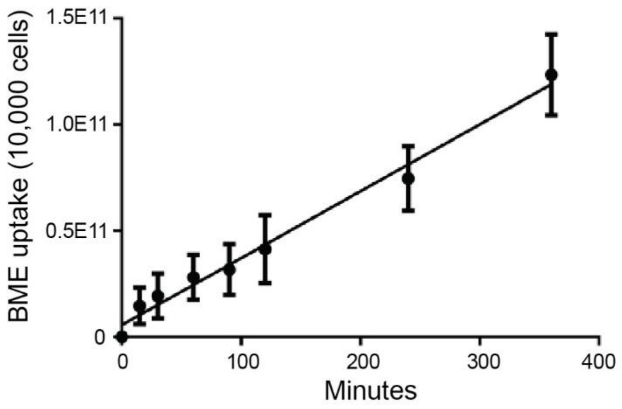

Supplementary Fig. S2. Time course of BEVs uptake by bEnd.3 cells. BEVs were labeled with FM4-64 (*n* = 3).

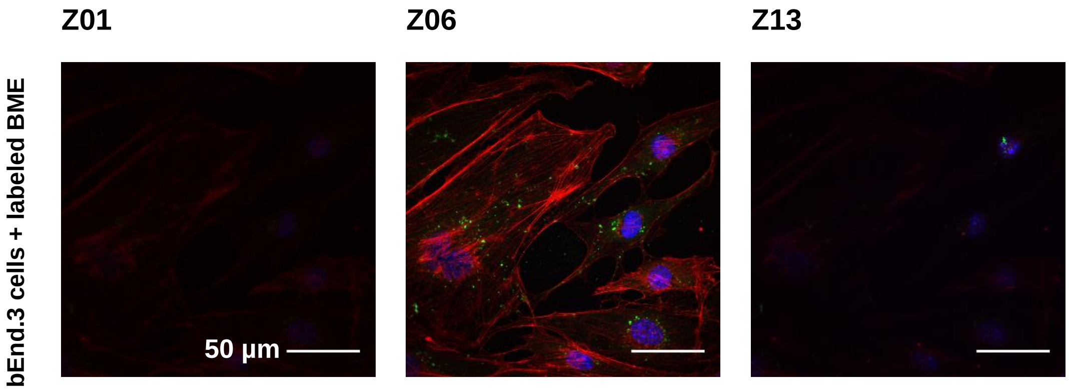

Supplementary Fig. S3. BEVs enter the interior of bEnd.3 cells. Images represent select focal planes acquired by Z-stack confocal microscopy. mRNA in BEVs was labeled with ExoGlow-RNA^{TM} (green). Nuclei and actin (cytoplasm) were stained with DAPI (blue) and Alexa Fluor 568 phalloidin (red), respectively. Merged images are shown. Magnification = 60X.

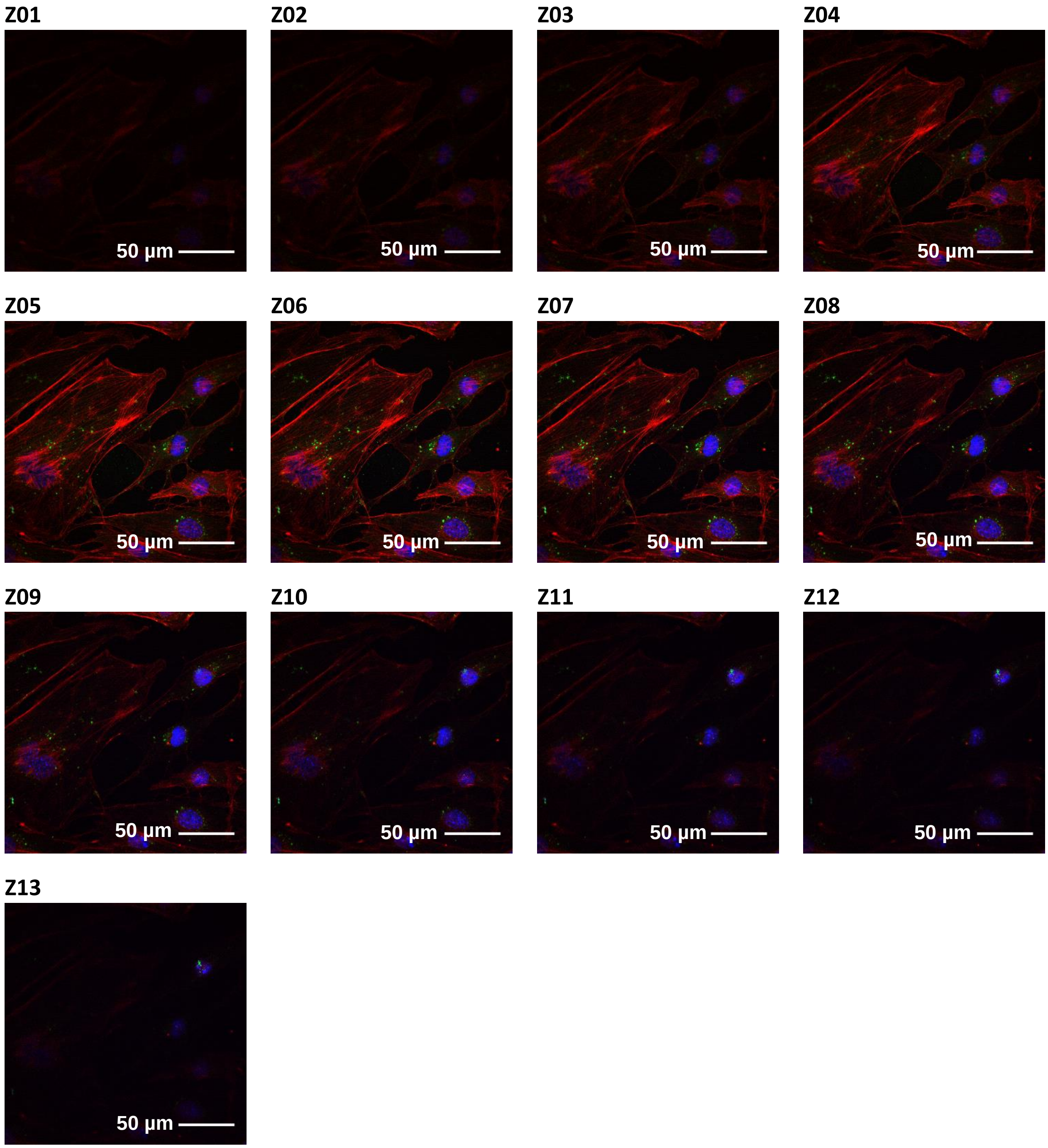

Supplementary Fig. S4. Overlay of individual Z-stack slices for bEnd.3 cells treatment group. Cells were incubated with BEVs in which mRNA was labeled with ExoGlow-RNA^{TM} (green) for 24 h. Nuclei and actin (cytoplasm) were stained with DAPI (blue) and Alexa Fluor 568 phalloidin (red) respectively. Magnification = 60X.

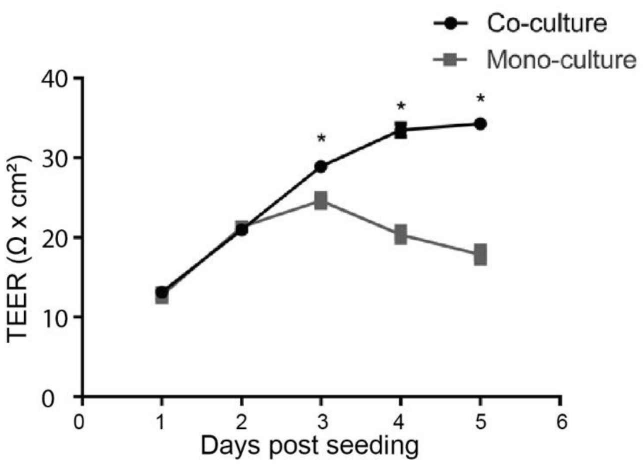

Supplementary Fig. S5. Daily transepithelial electrical resistance (TEER) measurements of bEnd.3 monocultures and co-cultures with C8-D1A astrocytes.

\

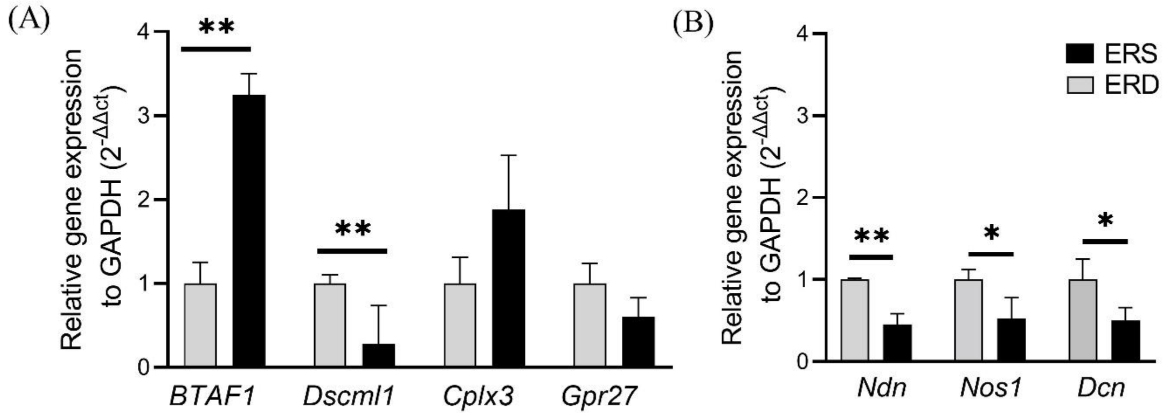

Supplementary Fig. S6. qRT-PCR validation of seven differentially expressed genes identified by RNA-seq analysis in left hippocampus from C57BL/6J mice fed ERS diet compared with ERD diet in male **(A)** and female **(B)**. Values are means ± SEMs. *P<0.05, **P<0.01, n = 3.

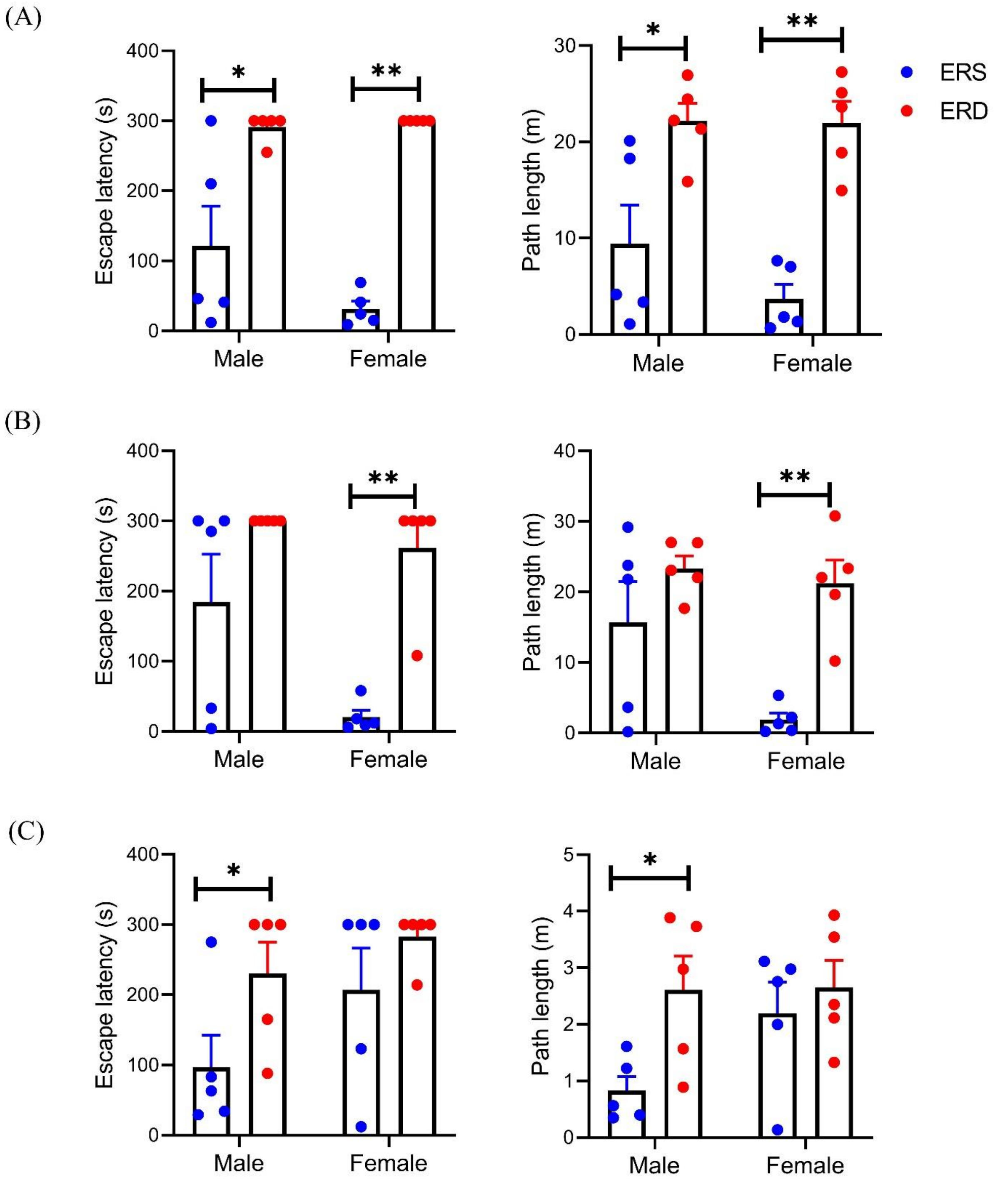

Supplementary Fig. S7. Effects of BEV-defined diets on spatial learning and memory in C57BL/6J mice ages 4 weeks **(A)**, 7 weeks **(B)** and 15-18 weeks **(C)**. *P<0.05, **P<0.01 by Mann-Whitney test (n = 5 per sex and age).

Supplementary Table 1. qRT-PCR primers.

Gene	Primer (5'→ 3')	Reference
Cplx3	F: GAGAAGAGTACGAGGAGTATCAG<br>R: CAGCTGGATCTGGCTTTCATCT	[40]
BTAF1	F: GCCTTTGGAAAGCTTTTGTG<br>R: CCAGTACCTGCCCCATGT	[41]
Gpr27	F: GAAGAGGCTGTGCAAGATGTT<br>R: AGCTCCCGGTTGAAGAGGA	[42]
Dscaml1	F: AGGCTGAAGAGGCTACGAGA<br>R: GAGGTCCTTTCACAGGGGTG	[43]
Ndn	F: GTATCCCAAATCCACAGTGC<br>R: TAACTCTCCAGGGCCTTCTT	[44]
Nos1	F: ATCTGTCTCGCCAGCCATCAGCCA<br>R: GGAGCTTTGTGCAGTTTGCCGTCG	[45]
Dcn	F: CTGGGCTGGCACAGCATAAGTA<br>R: CGGACAGGGTTGCCGTAAAG	[46]
GAPDH	F: TGTTCCTACCCCCAATGTGT<br>R: TGTGAGGGAGATGCTCAGTG	[47]

F, forward. R, reverse.

Supplementary Table 2. Racine scale scores of C57BL/6J mice ages 21 weeks fed small extracellular vesicles (sEVs) and RNA-defined diets starting at weaning.

Diet (sex)	Score								Mean[2]	SEM
	0	1	2	3	4	5	6	7		
ERS (male)	7	1							0.13	2.12
ERD (male)	4	3	1						0.63**	0.72
ERS (female)	1	3			1	1	1	1	3.13	0.30
ERD (female)		2		1	2	1		2	4	0.22

[1]The revised Racine scale reports scores for the time after kainic acid administration when effects of treatment were largest (20 minutes for males, 120 minutes for females).

[2]Mean scores were calculated by multiplying the number of mice in each category with the numerical score in that category and dividing that number by the number of animals in the treatment group ($n = 8$). $^{**}P < 0.01$ by two-way ANOVA analysis (time *x* diet) of log transformed data.
